# Regulation of APD and Force by the Na^+^/Ca^2+^ Exchanger in Human-Induced Pluripotent Stem Cell-Derived Engineered Heart Tissue

**DOI:** 10.3390/cells11152424

**Published:** 2022-08-05

**Authors:** Djemail Ismaili, Katrin Gurr, András Horváth, Lei Yuan, Marc D. Lemoine, Carl Schulz, Jascha Sani, Johannes Petersen, Hermann Reichenspurner, Paulus Kirchhof, Thomas Jespersen, Thomas Eschenhagen, Arne Hansen, Jussi T. Koivumäki, Torsten Christ

**Affiliations:** 1 Institute of Experimental Pharmacology and Toxicology, University Medical Center Hamburg-Eppendorf, 20246 Hamburg, Germany; 2 Department of Cardiology, University Heart & Vascular Center Hamburg, University Medical Center Hamburg-Eppendorf, 20246 Hamburg, Germany; 3 DZHK (German Centre for Cardiovascular Research), Partner Site Hamburg/Kiel/Lübeck, 20246 Hamburg, Germany; 4 Department of Biomedical Sciences, University of Copenhagen, 2200 Copenhagen, Denmark; 5 Department of Cardiovascular Surgery, University Heart and Vascular Center, 20246 Hamburg, Germany; 6 Institute of Cardiovascular Sciences, College of Medical and Dental Sciences, University of Birmingham, Birmingham B15 2TT, UK; 7 BioMediTech, Faculty of Medicine and Health Technology, Tampere University, 33520 Tampere, Finland

**Keywords:** hiPSC-CM, human ventricular cardiomyocytes, rat ventricular cardiomyocytes, NCX, APD, force, SEA0400

## Abstract

The physiological importance of NCX in human-induced pluripotent stem cell-derived cardiomyocytes (hiPSC-CMs) is not well characterized but may depend on the relative strength of the current, compared to adult cardiomyocytes, and on the exact spatial arrangement of proteins involved in Ca^2+^ extrusion. Here, we determined NCX currents and its contribution to action potential and force in hiPSC-CMs cultured in engineered heart tissue (EHT). The results were compared with data from rat and human left ventricular tissue. The NCX currents in hiPSC-CMs were larger than in ventricular cardiomyocytes isolated from human left ventricles (1.3 ± 0.2 pA/pF and 3.2 ± 0.2 pA/pF for human ventricle and EHT, respectively, *p* < 0.05). SEA0400 (10 µM) markedly shortened the APD_90_ in EHT (by 26.6 ± 5%, *p* < 0.05) and, to a lesser extent, in rat ventricular tissue (by 10.7 ± 1.6%, *p* < 0.05). Shortening in human left ventricular preparations was small and not different from time-matched controls (TMCs; *p* > 0.05). Force was increased by the NCX block in rat ventricle (by 31 ± 5.4%, *p* < 0.05) and EHT (by 20.8 ± 3.9%, *p* < 0.05), but not in human left ventricular preparations. In conclusion, hiPSC-CMs possess NCX currents not smaller than human left ventricular tissue. Robust NCX block-induced APD shortening and inotropy makes EHT an attractive pharmacological model.

## 1. Introduction

The development of human-induced pluripotent stem cell-derived cardiomyocytes (hiPSC-CMs) is expected to become a relevant step for the translational facet of cardiovascular basic research. HiPSC-CMs provide human disease models, enabling the detailed functional study of patient-specific hiPSC-CMs in a dish [1,2]. Nevertheless, there is concern that hiPSC-CMs may not fully reflect human heart physiology.

Ca^2+^ signaling is crucial for proper heart function and defective intracellular calcium signaling is a key process driving heart failure, arrhythmias, and vascular dysfunction, but may differ in hiPSC-CMs that lack the highly organized expression of the electromechanical coupling components. HiPSC-CMs have a less well developed t-tubular network, and their sarcomeric structures are less organized than those of adult cardiomyocytes (CMs) [3]. Despite these morphological differences, a recent report suggests that Ca^2+^ signaling in hiPSC-CMs appears to resemble that of rabbit ventricular CMs [4]. Authors used established and sophisticated methods to measure calcium influx via Ca^2+^ channels, Ca^2+^ cycling via sarcoplasmic reticulum, and Ca^2+^ extrusion via a sodium calcium exchanger (NCX). However, as the experimental conditions necessary for performing such studies often are far from the physiologically relevant situation, a comparison with in vivo calcium regulation may be questionable. Furthermore, results are difficult to interpret and have resulted in some controversy [5].

Here, we used a different approach. We directly measured NCX currents using an established patch clamp protocol [6]. To estimate the NCX contribution to the force and action potential duration (APD), we applied the NCX blocker SEA0400 in hiPSC-CMs cultured as engineered heart tissue (EHT). Importantly, the hiPSC-CM results were compared with the data from rat and human left ventricular tissue, obtained under comparable experimental conditions.

## 2. Methods

### 2.1. Rat Tissue

The study was conducted according to guidelines for laboratory animal welfare of the National Institutes of Health (publication no. 85-23, revised 1985). All experiments with rat tissue were performed in compliance with the German Law for the Protection of Animals. Moreover, 160 to 240 g male Wistar rats (Charles River Laboratories, Wilmington, MA, USA) were decapitated under light CO_2_ anesthesia (70% CO_2_ and 30% O_2_) and the excised hearts were placed in oxygenated and modified Tyrode’s solution at 37°C containing (in mM): NaCl—126.9, KCl—5.4, CaCl_2_—1.8, MgCl_2_—1.05, NaHCO_3_—22, NaH_2_PO_4_—0.45, EDTA—0.04, ascorbic acid—0.2, pyruvate—5, and glucose—5. For contractility and action potential measurements, small ventricular chunks were prepared from ventricular tissue. For patch clamp measurements, rat CMs were isolated via enzymatic dissociation using collagenase type 1 (254 U/mL; Worthington Biochemical, Lakewood, NJ, USA) on a Langendorff apparatus, as described earlier [7].

### 2.2. Human Tissue

The study followed the declaration of Helsinki and was approved by the ethics committee of the Medical Council of Hamburg, Germany (approval number: OB-088/04 and PV3759). Human left ventricular free wall tissue samples were obtained with informed consent from 13 patients undergoing implantation of the left ventricular assist device, heart transplantation, or aortic valve surgery at the University Heart and Vascular Center Hamburg. The patients were 53.7 ± 4.3 years old and left ventricular ejection fraction was 27.5 ± 4.7%. Further patient characteristics are outlined in Table S1. After the excision of ventricular tissue, samples were transferred in cardioplegic solution to the laboratory. The composition of cardioplegic solution in mM: NaCl—100, taurine—50, glucose—20, KCl—10, MgSO_4_—5, MOPS (3-(N-morpholino) propanesulfonic acid)—5, KH_2_PO_4_—1.2, and 2.3-butanedione monoxime (BDM)—30. For patch clamp measurements, human CMs were isolated via enzymatic dissociation using protease type XXIV (5 U/mL; Sigma, St. Louis, MO, USA) and collagenase type 1 (254 U/mL; Worthington Biochemical, Lakewood, NJ, USA), as described previously in detail [8].

### 2.3. Human-Induced Pluripotent stem cell-Derived Engineered Heart Tissue

Ventricular-like hiPSC-CMs were differentiated from an in-house control hiPSC cell line [9]. Differentiated hiPSC-CMs were used to generate three-dimensional EHT (1 × 10^6^ cells/100 μL EHT). The expansion and differentiation of hiPSC-CMs and the generation of EHT were performed according to published in-house standardized protocol [10]. EHT was cultured for 24–29 days under identical conditions at 37 °C in a 7% CO_2_ and 40% O_2_humidified cell culture incubator with a medium consisting of DMEM (Biochrom, Berlin, Germany), 10% heat-inactivated horse serum (Gibco, Paisley, Scotland), 1% penicillin–streptomycin (Gibco, Paisley, Scotland), insulin (10 μg/mL; Sigma, St. Louis, MO, USA), and aprotinin (33 μg/mL; Sigma, St. Louis, MO, USA). After culturing, hiPSC-CMs applied for patch clamp measurements were isolated with collagenase II (200 U/mL; Worthington Biochemical, Lakewood, NJ, USA) for 5 h. Isolated cells were plated on gelatine-coated (0.1%) glass coverslips (12 mm diameter; Carl Roth GmbH + Co, Karlsruhe, Germany) and kept in culture for 24–48 h to maintain adherence under superfusion in the recording chamber during patch clamp measurements.

### 2.4. Patch Clamp Measurements

**Figure 1:**
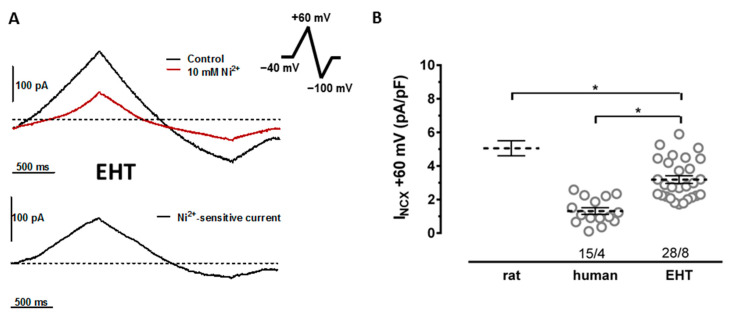
NCX currents in cardiomyocytes from rat ventricle, human ventricle, and EHT culture. (**A**) NCX current traces elicited by a ramp pulse from a holding potential of −40 mV to +60 mV, then to −100 mV, and back to the holding potential of −40 mV (see inset) at the pre-drug control (black trace) and after superfusion of cells with 10 mM Ni^2+^ (dark red trace). (**B**) NCX current density in the presence of 10 mM Ni^2+^ given as individual data points (circles) and respective mean values ± SEM measured in CMs from human ventricle and EHT culture. For comparison of mean ± SEM NCX currents measured in rat ventricular, CMs from Christ et al. [7] are given. n/n indicates the number of cells/number patients for human and the number of cells/number of isolations for EHT, * indicates significance.

The whole-cell configuration of the single-electrode patch clamp technique was used to record membrane current measurements with the Axopatch 200B (Axon Instruments, Foster City, CA, USA). Cells were investigated in a recording chamber (RC-26G; Warner Instruments, Hamden, CT, USA) placed on the stage of an inverted microscope (Axiovert 40 CFL; Zeiss, Oberkochen, Germany) at 37 °C. Heat-polished pipettes were pulled from borosilicate-filamented glass (Hilgenberg, Malsfeld, Germany) using the DMZ Universal Puller (Zeitz Instruments GmbH, Martinsried, Germany). Tip resistances were 2–5 MΩ and seal resistances were 3–6 GΩ. The data were acquired and analyzed using the ISO2 software (MFK, Niedernhausen, Germany). To measure the NCX current, we applied previously described electrophysiological protocols [6]. In brief, we used K^+^-free bath and pipette solutions. The composition of bath solutions in mM: NaCl—135, CsCl—10, CaCl_2_—1, MgCl_2_—1, BaCl_2_—0.2, NaH_2_PO_4_—0.33, TEACl—10, HEPES—10, glucose—10, pH adjusted to 7.4 with NaOH. The solution contained (in mM) lidocaine (0.05), nisoldipine (0.001), and ouabain (0.02) to block the Na^+^, Ca^2+^, and Na^+^/K^+^ pump currents. The composition of pipette solutions in mM: CsOH—140, aspartic acid—75, TEA-Cl—20, Mg^2+^-ATP—5, HEPES—10, NaCl—20, EGTA—20, CaCl_2_—10, pH adjusted to 7.2 with CsOH. The cell capacitance was calculated from the current response to a 5 mV voltage step and was than compensated for measuring the membrane current. The currents were measured in a constant flow (2 mL/min) low-volume chamber (300 µL) through ramp pulses (frequency 0.1 Hz), initially leading to depolarization from the holding potential of −40 mV to 60 mV, followed by hyperpolarization to −100 mV, and depolarization back to the holding potential of −40 mV (Figure 1). The NCX current was defined as the Ni^2+^-sensitive current measured by application of 10 mM NiCl_2_.

### 2.5. Action Potential Measurements

The action potentials (APs) in rat and human ventricular tissue and EHT were recorded with standard sharp microelectrodes, as previously described [7,11]. In brief, both ventricular tissue and EHT were pinned down in a recording chamber and field-stimulated by two platinum wires at 1 Hz (unipolar pacing, 0.5 ms stimulus duration, 50% above the stimulation threshold). Sharp microelectrodes were pulled from borosilicate glass (Hilgenberg, Malsfeld, Germany) using the DMZ Universal Puller (Zeitz-Instruments GmbH, Martinsried, Germany). The tip resistances ranged from 20 to 50 MΩ when filled with 3 M of KCl. The signals were amplified with a BA-1s npi amplifier (npi electronic GmbH, Tamm, Germany). The APs were recorded and analyzed using the Lab-Chart software (AD Instruments Pty Ltd., Castle Hill NSW, Australia). The compound effects were measured after an equilibration period of 30 min. In order to calculate the AP parameters, five consecutive APs were averaged.

### 2.6. Contractility Measurements

To measure the contractile force in rat and human ventricular muscle, strip preparations were mounted in organ baths filled with 50 mL of Tyrode solution, as described previously [7]. Rat preparations were stretched to 5 mN [12]. Human preparations were pre-stretched to a 50% length, giving the maximum force, and paced at a frequency of 1 Hz with 5 ms electrical pulses 10% above the threshold intensity. The tissue samples were pretreated for 90 min with the irreversible α-adrenoceptor blocker phenoxybenzamine (6 µM) [13]. The compound effects were measured after an equilibration period of 30 min. The recordings were analyzed using Chart software (AD Instruments Pty Ltd., Castle Hill NSW, Australia). Force data were not normalized to the muscle square area [14].

To measure the contractile force in EHT, a video-optical contractility test system (EHT Technologies GmbH, Hamburg, Germany) was applied, as described previously [15]. Briefly, video files generated by a software-based automated video-optical recording system were analyzed by deflecting the silicon posts during EHT contraction. Based on the EHT shortening during contractions, force and kinetic parameters were calculated using CTMV software (CTMV GmbH & Co. KG, Pforzheim, Germany).

### 2.7. Computational Simulations with Rat, Human and hiPSC Cardiomyocyte Models

Computational modelling and simulations were performed using recently published, yet established, rat ventricular [16] and human ventricular [17] CM models. For the hiPSC-CM model, we used the same approach as previously described [11,18]. Briefly, hiPSC-CM model version was obtained by increasing the calcium current (I_Ca_) density 1.5-fold and increasing the density of the rapidly activating potassium current (I_Kr_) density 4-fold, as well as decreasing the inwardly rectifying potassium current density (I_K1_) 0.5-fold in the human ventricular CM model [17]. For the sake of simplicity, we omitted the T-type calcium current from this implementation, as its role was found insignificant to excitation–contraction coupling in our previous study [11]. As suggested by the experiments presented in this study, we extended the hiPSC-CM model version to include 2.5-fold stronger NCX current. As the EHT experiments were carried out using an external pacing stimulus current, we excluded the pacemaking current, I_f_, from the hiPSC-CM model implementation, similar to our earlier approach [11].

The gradual NCX block in silico experiments were simulated by multiplying the parameter that defines the maximum NCX rate with the corresponding value constant. In all cases, in silico results were obtained by running the CM models first to the steady-state control at 1 Hz pacing, switching the NCX block parameter, and finally running the model to the new drug-induced steady state. The data from one AP cycle were recorded from each of those steady states for plotting and further analysis.

By readjusting the sodium–calcium exchanger plasma membrane Ca^2+^ ATPase (NCX-PMCA), the Ca^2+^ extrusion ratio rat ventricular and human ventricular CM models were carried out via simple hand-tuning, as it was deemed sufficient for the purpose of our hypothesis testing. In the case of the rat ventricular CM model, the maximum exchange rate of NCX was decreased by 20% and maximum pump rate of plasma membrane Ca^2+^ ATPase (PMCA) was increased by 50%. To reset the sarcoplasmic reticulum [Ca^2+^] to the original range, the conductance of background Ca^2+^ current was decreased by 20%. In the case of the human ventricular CM model, a slightly larger set of tuning parameters was needed, because the PMCA pump was so weak in the original model version [17] that it hardly made any impact on Ca^2+^ extrusion. To fix this, the PMCA pump rate was increased 740-fold (from 5 × 10^−4^ to 0.37). The maximum exchange rate of NCX was decreased by 60%. Furthermore, we decreased the maximum permeability of the L-type Ca^2+^ channel by 33% to reset the sarcoplasmic reticulum [Ca^2+^] to the original range. As the modifications needed for readjusting the NCX-PMCA ratio were more dramatic for the human ventricular CM model, the additional tuning of K^+^ current conductances was needed to reset the APD to the original range. We accomplished this using the global multiplier with a value of 0.73 to reduce the maximum conductance of all K^+^ currents by 27%.

The EC_50_ values for SEA0400 blocking NCX lie between 2.2 µM in the HEK cells expressing human NCX [19] and 780 nM in the human atrial CMs [7]. From these findings, we would expect between 82 and 92% of the NCX block to use 10 µM SEA0400. To cover that range, we ran the models with 50, 70, and 90% of the NCX block.

### 2.8. Drugs

SEA0400 (2-[4-[(2.5-difluorophenyl)methoxy]phenoxy]-5-ethoxyaniline) was synthesized in the Institute of Pharmaceutical Chemistry, University of Szeged (Szeged, Hungary). The stock solutions were prepared in 100% DMSO and stored at −20 °C. BaCl_2_ and NiCl_2_ were obtained from Calbiochem (Merck, Darmstadt, Germany). All other drugs and chemicals were obtained from Sigma (St. Louis, MO, USA).

### 2.9. Statistics

Statistical analyses and curve fitting were performed using GraphPad Prism software version 7 (GraphPad Software, San Diego, CA, USA). The data were compared using Student’s unpaired or paired *t* test or 1-way ANOVA, where appropriate. Differences with a value of *p* < 0.05 were considered statistically significant. Data are presented as mean ± SEM.

## 3. Results

### 3.1. NCX Currents in hiPSC-CMs Are Not Smaller Than in Human Ventricular Cardiomyocytes

First, we measured mRNA expression for the NCX isoforms NCX1-3 (genes: *SLC8A1*, *SLC8A2* and *SLC8A3*) [20]. The mRNA for all three isoforms of NCX was expressed in the human left ventricle (LV) and also in EHT. Expressions for NCX1, the dominant isoform, were slightly but significantly higher in EHT vs. human LV (Supplementary Figure S1).

The NCX currents were measured in isolated CMs. In order to facilitate comparisons to the literature, we used voltage ramps, which have been frequently used in previous studies [6]. The NCX currents were defined as Ni^2+^-sensitive currents evoked during voltage ramp protocols. We found NCX currents in each single cell from all four batches of hiPSC-CMs cultured from EHT (Supplementary Figure S2). In EHT, the NCX currents were larger than in the human left ventricular CMs (Figure 1). However, the NCX current densities in EHT did not reach the level recently reported by us for rat left ventricular CMs studied using the same protocol [7].

### 3.2. NCX Block Shortens the APD in EHT but Not in the Human Left Ventricle

**Figure 2:**
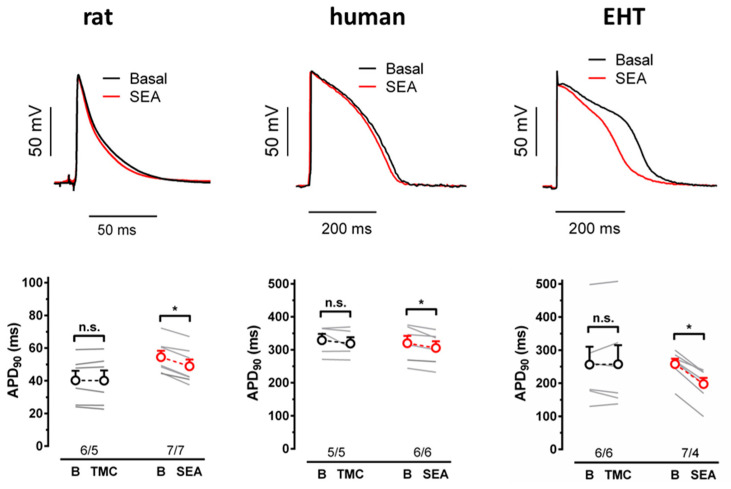
Action potentials in rat ventricle, human ventricle, and EHT. (**Up**) Action potential traces at pre-drug control (black trace) and after 20 min superfusion with 10 µM SEA0400 in rat ventricle, human ventricle, and EHT. (**Bottom**) Individual data points (light gray) and respective mean values ± SEM for APD_90_ given as time-matched controls (TMCs) and in the presence of 10 µM SEA0400 in rat ventricle, human ventricle, and EHT. n/n indicates the number of cells/number rats or humans and the number of cells/number of isolations for EHT, * indicates significance, n.s. indicates non-significance.

During the plateau phase of the APs, NCX should mediate a depolarizing current [21]. Thus, the block of NCX should shorten the APD. Therefore, we measured the effects of the acute block of NCX by SEA0400 on the APD in EHT and in the left ventricular tissue from humans and rats. In all three tissues, we did not see significant changes in the APD in time-matched controls (TMCs) over 30 min (Figure 2). In contrast, the APD_90_ was markedly shortened by SEA0400 (10 µM) in EHT (by 24.6 ± 3.5%). In the left ventricular papillary muscles of rats, SEA0400-induced shortening of the APD_90_ was significantly smaller than in EHT, but still significant compared to TMCs (by 10.7 ± 1.6%). In left ventricular preparations from humans, SEA0400 tended to shorten the APD_90,_ but the apparent effect size was not larger than the non-significant trend in TMCs (by 4.4 ± 1.1% vs. 2.9 ± 2.5% in TMCs).

### 3.3. SEA0400 Does Not Block Calcium Currents in hiPSC-CMs

SEA0400 shortened the APD_90_ in EHT, but not in the human LV compared to TMCs. This finding may imply that SEA0400 non-selectively blocks other depolarizing currents in hiPSC-CMs, but not in the human LV. However, the action potential amplitude (APA) or maximum upstroke velocity were not affected (in the rat and human LV or in EHT, Table S2), arguing against the relevant block of Na^+^ currents using SEA0400. HiPSC and human CMs differ with respect to Ca^2+^ channels. While human ventricular CMs express only L-type Ca^2+^ channels, T-type Ca^2+^ channels contribute substantially to the total Ca^2+^ currents in hiPSC-CMs [22]. Therefore, we measured the effects of SEA0400 on I_Ca_ in hiPSC-CMs. As shown before [22], there was a slight, but still significant, decrease in I_Ca_ in TMCs. More importantly, the decrease was not larger in hiPSC-CMs exposed to 10 µM of SEA0400 (Supplementary Figure S3).

### 3.4. NCX Block Increases Force in EHT but Not in the Human Left Ventricle

**Figure 3:**
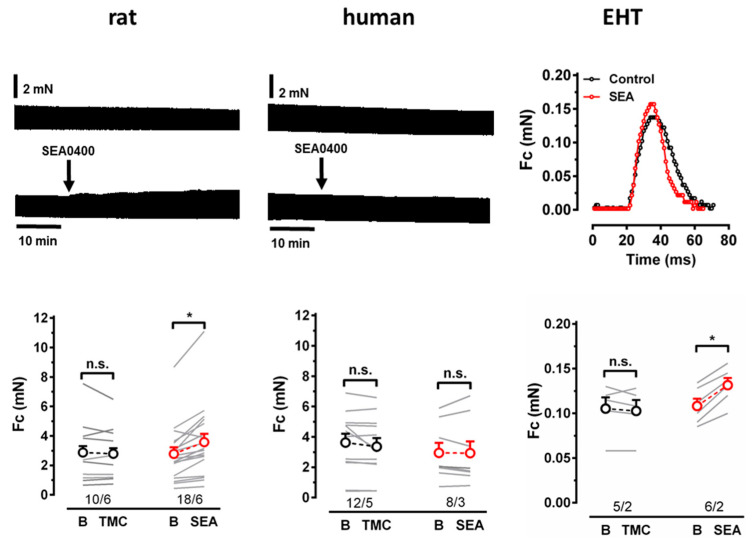
Force measurements in rat ventricle, human ventricle, and EHT. (**Up**) Time course of force in rat and human ventricle given as time-matched controls (TMCs) and exposed to 10 µM SEA0400. Single contraction of force in EHT given as TMCs and exposed to 10 µM SEA0400. (**Bottom**) Individual data points (light gray) and respective mean values ± SEM for force given as time-matched controls (TMCs) and in the presence of 10 µM SEA0400 in rat ventricle, human ventricle, and EHT. n/n indicates the number of cells/number rats or humans and the number of cells/number of isolations for EHT, * indicates significance, n.s. indicates non-significance.

NCX is believed to represent one of the main Ca^2+^-extruding mechanisms; therefore, the block of NCX should increase intracellular Ca^2+^ and consecutively increase the force generation [21]. We measured the effects of the acute NCX block with SEA0400 on the force in EHT and in LV preparations from rats and humans. In the human LV and EHT, the force in time-matched controls declined slowly over 30 min (Figure 3). In EHT, SEA0400 (10 µM) significantly increased the force (to 22.3 ± 3.3% of pre-drug control values). In contrast to the human LV, SEA0400 did not show any consistent effect on force (−3.7 ± 4.3% vs. −6.2 ± 3.7% in TMCs). For comparison, we measured the effects of the compound in intact rat ventricular papillary muscles. The force remained stable over 30 min in TMCs, but increased significantly upon SEA0400 exposure (to 31 ± 5.4% of pre-drug control values).

### 3.5. Effects of NCX Block on AP and Force in Comparison to Computational Model Predictions

To better understand whether the effects of SEA0400 on the APD and force are in line with common paradigms on NCX contribution to Ca^2+^ handling in human ventricle, we employed established computational models for rat [16] and human [17] ventricular CMs, as well as for hiPSC-CMs. Unfortunately, in the initial simulations, in which the original model versions were used, the NCX block turned out to be an intolerable condition, leading to intracellular Ca^2+^ overload and consequent AP abnormalities. For the rat CM model, a 50% NCX block was already too great of a challenge (Supplementary Figure S4). In contrast, the human CM model was a bit more robust, tolerating NCX blocks up to 70% (Supplementary Figure S5).

**Figure 4:**
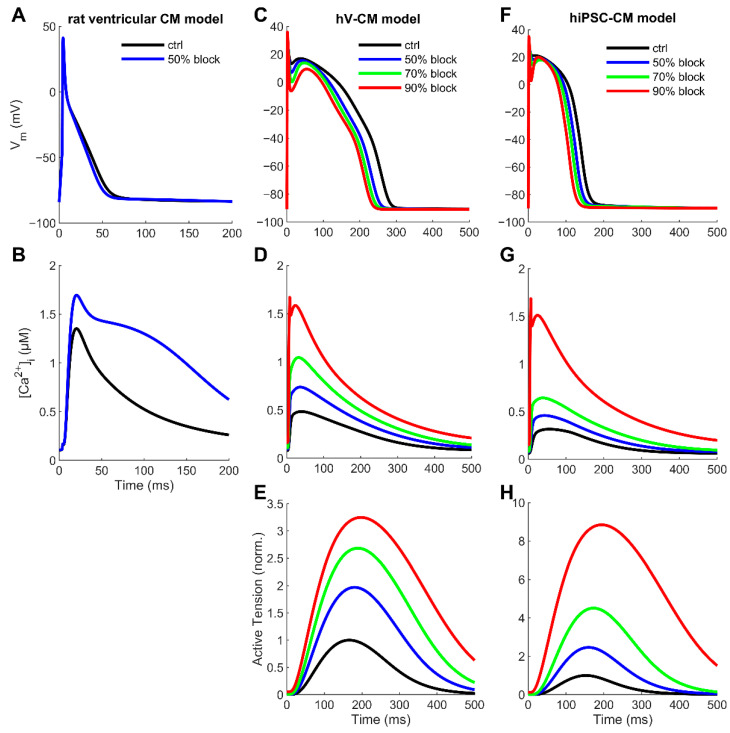
Simulated effect of NCX block in the rat ventricular, human ventricular, and hiPSC cardiomyocyte models. The principal outputs: membrane voltage (**A**,**C**,**F**), calcium transient (**B**,**D**,**G**), and active tension (**E**,**H**) with different degrees of NCX block. For details of the used models and simulation protocols, please see the Methods section.

We hypothesized that the standard models may underestimate the Ca^2+^ extrusion using the PMCA, likely causing a lack of robustness. It is generally assumed that, in murine CMs, sarcoplasmic reticulum Ca^2+^ ATPase (SERCA) pumps ~90% of Ca^2+^ back to the SR and NCX extrudes ~10%, while the PMCA contributes 1% or less [23]. However, there is substantial experimental evidence that the PMCA can remove Ca^2+^ at a rate of 30% of that of NCX [24,25,26]. When we readjusted the rat CM model accordingly, it became a bit more robust, capable of handling a 50% NCX block (Supplementary Figure S4). The simulated drug impact resulted in a 11.1% reduction in the APD_90_ (Figure 4A). This is in the same range as the SEA0400-induced shortening of APs in the experiments. However, the simulated block of NCX is only 50%, compared to the ~90% block to be expected in the experiments, so the conditions are not fully comparable.

To our knowledge, a thorough analysis of SERCA-NCX-PMCA Ca^2+^ removal fractions for human ventricular CMs has not been published. However, there are several data sets, from which these values can be integratively estimated (please see Supplementary Computations for details). When we readjusted the human CM model accordingly, it functioned a bit more robustly, being able to also handle a 90% NCX block. The simulated impact of the 90% NCX block on the APD_90_ (−18.3%) was much greater than the tiny non-significant change observed in the experiments. However, the effect was substantially decreased compared to the original model (by 34.9%), as shown in Supplementary Figure S5A. Similarly, the great increase in force due to the NCX block (Figure 4E) in simulation does not match the experimental observation of no change.

We also repeated the same NCX block simulations with a hiPSC-CM model that is described in detail in the Methods section. Interestingly, the model prediction for AP shortening due to the NCX block (APD_90_ −22.8%) fits perfectly with the experimentally observed change (−26.6 ± 5%). However, the simulated increase in force (almost nine-fold; Figure 4H) is far greater than in the experiments (+20.8 ± 3.9%). This indicates that even with a readjusted NCX-PMCA Ca^2+^ extrusion ratio, the computational CM model is not robust enough to resist intracellular Ca^2+^ accumulation, when NCX function is almost fully blocked.

## 4. Discussion

In our study, we compared the densities of the NCX current in hiPSC-CMs to ventricular CMs from rat and human and estimated the NCX contribution to AP and force. The main finding is that NCX currents in hiPSC-CMs from EHT are larger than in the human LV. Furthermore, the block of NCX evokes measurable effects on the APD and force in EHT and in the LV from rat but not from humans.

### 4.1. NCX Current Density

We found slightly higher transcript abundances for NCX1 in EHT vs. the human LV (Supplementary Figure S1), which were associated with substantially larger NCX currents in EHT, indicating relevant differences in functional channel-forming proteins. Interestingly, the ~2.4-fold larger NCX in EHT fits closely to the ~2 fold larger I_Ca_ in EHT vs. the human LV (12 pA/pF vs. 6 pA/pF) [22,27], and could thereby be interpreted as a balanced activity between transsarcolemmal Ca^2+^ load and Ca^2+^ extrusion. In rat ventricular myocytes, the NCX current was much higher than in EHT, despite the fact that I_Ca_ did not substantially differ (10.2 pA/pF) [28]. A much higher ratio of NCX/I_Ca_ is likely needed to extrude Ca^2+^ during shorter APs.

### 4.2. Contribution of NCX to the APD in Heart Muscle

As expected, SEA0400 shortened the APD both in rat ventricular tissue and in EHT. Even though basal APD_90_ values widely differ between the two tissues, the effect size was clearly stronger in EHT than in rats, both absolutely and relatively. This indicates that NCX is more important for the regulation of repolarisation in human EHT than in rats. As for force, we cannot provide an explanation for the lack of SEA0400 effects on the APD in the human LV. Results are in line with several papers demonstrating an absence of APD shortening by the NCX block in ventricular tissue from dogs, closely resembling the electrophysiology of the human heart [29,30,31]. Furthermore, our results are in line with findings on a new (even more selective than SEA0400) NCX blocker that did not shorten the APD in human left ventricular preparations but in hiPSC-CMs [19]. Thus, the data suggest fundamental differences in NCX contribution to Ca^2+^ handling, and hence APD regulation between hiPSC-CMs and the mature human LV.

### 4.3. Contribution of NCX to Force in Heart Muscle

The first NCX blockers such as KB-R7943 showed poor selectivity [21]. Thus, there was a huge interest in knock-out (KO) models of NCX. Unexpectedly, NCX1 KO mice showed regular Ca^2+^ transients [32], indicating that chronic adaptation can compensate for the lack of NCX function in the KO animals. While Ca^2+^ cycling via sarcoplasmic reticulum remained unchanged in NCX-KO mice, the I_Ca_ density was smaller, thereby reducing Ca^2+^ entry and subsequently the need to extrude Ca^2+^ via NCX. In addition, mice may not be the species of choice to study human NCX physiology, since NCX contributes to the regulation of [Ca^2+^]_i_ predominantly during the APs, which is much shorter in mice than in humans.

Nevertheless, positive inotropy upon the acute pharmacological NCX block could be consistently demonstrated in rat ventricular tissue. Our findings in isolated rat ventricular tissue are in line with data obtained in Langendorff-perfused rat hearts [33]. With this in mind, one would expect a larger positive inotropic effect by the NCX block in species with a longer APD, but SEA0400 was devoid of positive inotropy in guinea pig hearts (APD ~150–200 ms) [34,35] and in human LV tissue (this study). Comparing the size of the positive inotropic effects in rat ventricular tissue vs. EHT is difficult because the dynamic range of force regulation differs. In rat ventricular tissue, catecholamines and/or high concentrations of calcium increase force by ~5 mN (the same experimental conditions) [28]. The increase in force using the NCX block amounts to about 20% of maximum force. In contrast, the maximum force increases as high calcium in EHT only amounts to ~0.15 mN [36]. Thus, the positive inotropy using the NCX block in EHT reached a 60% maximum force in this model. This finding could be interpreted as evidence for a larger contribution of NCX to Ca^2+^ handling in EHT.

### 4.4. Effect of NCX Block on the APD and Force Are Not Compromised by SEA0400’s Selectivity

Unexpectedly, SEA0400 did not increase force in heart tissues from several animals (Table 1). One hypothesis proposes the nonselective block of I_Ca_ by SEA0400 that may have masked positive inotropy induced by less Ca^2+^ extrusion [37]; in fact, some papers reported the I_Ca_ block using SEA0400 [38,39,40]. However, others did not see an effect of SEA0400 on I_Ca_ [41] and two considerations argue against the assumption that the block of I_Ca_ could explain the unexpected lack of effect of SEA0400 in guinea pig and human (Table 1). First, the block of I_Ca_ by SEA0400 should aggravate (not level off) APD shortening. However, SEA0400 does not shorten the APD_90_ in the human LV (this study and also recently reported) [19], human atrium [7], and dog ventricle [29,30,31]. Second, in our study, a decline in I_Ca_ in hiPSC-CMs exposed to increasing concentrations of SEA0400 was not larger than in time-matched controls (Supplementary Figure S3). These data argue against the nonselective block of I_Ca_ by SEA0400 under our experimental conditions, under which the compound induced strong APD shortening.

### 4.5. Effects of NCX Block: Immature EHT vs. Mature LV

The results of this study may be perplexing. Both LV and EHT express NCX and possess robust NCX currents when using established patch clamp protocols. HiPSC-CMs that are clearly immature with respect of intracellular architecture show the effects predicted by computer models which integrate our current understanding of Ca^2+^ handling, but native LV tissue from patients does not. The reasons for this are unclear. One could argue that LV tissue was obtained from patients in end-stage heart failure with well-known remodeling of Ca^2+^ handling. However, with regards to heart failure, the contribution of sarcoplasmic reticulum is reported to decrease and the contribution of NCX increased [47,48]. The PMCA represents an alternative way to extrude Ca^2+^ [49]. Therefore, we readjusted the PMCA contribution to Ca^2+^ removal to fit our experimental findings, increasing its maximum pump rate 740-fold in the computational human ventricular CM model. Under this condition, APD shortening upon the NCX block was almost halved, but still remained substantial (Supplementary Figure S5), deviating from the experimental findings. We therefore changed the modeling conditions further and tested a purely speculative human ventricular CM model version. In addition to higher pump rate, the PMCA was altered to be electroneutral. Under this condition, APD shortening upon the NCX block was decreased only very slightly (Supplementary Figure S6). In summary, our comparison of experimental and simulation results suggests that the computational human ventricular CM model is likely missing some elements of autoregulatory mechanisms related to Ca^2+^ handling, as the NCX block leads to substantial intracellular Ca^2+^ accumulation and consequent AP shortening.

Methodological issues could be relevant. Tissue penetration by SEA0400 could be an issue. However, the compound was active in intact rat left ventricular tissue strips (both APD and force in this study). No data are available about the effects of SEA0400 on the APD in whole rat hearts, but it induced a strong tendency for a concentration-dependent shortening of the QT interval in Langendorff-perfused rat hearts [50]. In addition, SEA0400 increased LV dp/dt_max_ in Langendorff-perfused rat hearts [50]. The data argue against the idea that a lack of APD or force effects of NCX blockers in human heart preparations can be explained by a lack of tissue penetration.

## 5. Limitations of the Study

We measured SEA0400 effects in rat ventricular tissue at 1 Hz, much lower than the physiological heart rate in this species. However, we used this rate for comparison with human tissue. We used only one hiPSC-CM cell line for our study. Access to the human LV is limited. The success rate of AP measurements is much smaller than for force recording. Thus, we could have overlooked the small effects of the NCX block on the APD in the human LV.

Before EHT can be recommended or discarded as an experimental tool to study human ventricular NCX, other NCX functions such as a contribution to the generation of early afterdepolarizations [6,41] or Na^+^-driven inotropy should be studied in adult human ventricular tissue and in EHT in parallel.

## 6. Conclusions

The pharmacological block of NCX shortens the APD and increases force in rat ventricle, as expected from textbook knowledge. This finding may illustrate that simple but rapidly acting compounds can provide better insight in NCX physiology than slowly developing mice KO models, prone to chronic adaptation. The NCX block shows the predicted effects on the APD in hiPSC-CMs, but not in the human LV, despite the fact that transsarcolemmal Ca^2+^ influx via I_Ca_ and Ca^2+^ extrusion via NCX, estimated from patch clamp data, do not differ widely. Obviously, the current density does not necessarily correlate to NCX contribution to the APD and force regulation. The lack of effect of the NCX block in human ventricle is in line with several other studies in the hearts of larger animals and should therefore be taken seriously. Reasons for the NCX block insensitivity of human ventricle needs to be clarified before hiPSC-CMs can be used as a human model to study NCX. However, the marked effects of NCX inhibition in EHT indicate high sensitivity in preclinical drug screening. For this purpose, EHT appears to be suitable, even better than human myocardial tissue.

## Figures and Tables

**Table 1:** Literature research: the effect of NCX block on the APD_90_ and contractility parameters in different species.

Species	Cell Type	Substance	Concentration (in µM)	APD_90_	Peak CaT	Cs	Force	Reference
Dog	Ventricle	SEA0400	1	—	↔	—	—	Nagy et al. [41]
		SEA0400	1	—	↔	↔	—	Birinyi et al. [37]
		SEA0400	1	↓	↔	—	—	Bourgonje et al. [40]
		SEA0400	1	↔	↔	↔	—	Nagy et al. [29]
		ORM-10103	10	↔	↔	↔	—	
		GYKB-6635	1	↔	—	—	—	Geramipouretal. [31]
		ORM-10962	1	↔	↑	↑	—	Kohajda et al. [30]
		ORM-10962	1	—	↑	↑	—	Oravecz et al. [42]
Human	Ventricle	ORM-11372	10	↔	—	—	—	Otsomaa et al. [19]
	Atrium	SEA0400	10	↔	—	—	↔	Christ et al. [7]
	HiPSC	ORM-11372	0.1; 0.3	↓	—	—	—	Otsomaa et al. [19]
Rat	Ventricle	SEA0400	0.3	—	↑	↑	—	Acsai et al. [39]
		SEA0400	1	—	↑	—	—	Szentandrássy et al. [33]
Mouse	Ventricle	SEA0400	0.3; 1	—	↑	↑	—	Ozdemir et al. [43]
		SEA0400	1	—	↑	—	—	Bögeholz et al. [44]
		SEA0400	1; 10	↓	↑	↑	↑	Tanaka et al. [45]
Guinea pig	Ventricle	SEA0400	1	—	↔	—	—	Szentandrássy et al. [33]
		SEA0400	1	—	—	—	↔	Tanaka et al. [35]
		SEA0400	1; 10; 100	↔	—	—	—	Amran et al. [46]
		SEA0400	1	↔	—	—	↔	Namekata et al. [34]
Pig	ventricle	SEA0400	0.3; 1	—	↑	↑	—	Ozdemir et al. [43]

Legend: ↑ = increase; ↓ = reduction; ↔ = no effect; — = not measured.

## Data Availability

Not applicable.

## Supplementary Materials

The following supporting information can be downloaded at: https://www.mdpi.com/article/10.3390/cells11152424/s1, Figure S1: RNA expression of NCX; Figure S2: NCX currents in cardiomyocytes from human ventricle and EHT culture divided by different patients and different batches; Figure S3: Effect of SEA0400 on calcium current (I_Ca_) in EHT; Figure S4: Comparison of NCX block impact in the original and readjusted rat ventricular cardiomyocyte models; Figure S5: Comparison of NCX block impact in the original and readjusted human ventricular cardiomyocyte models; Figure S6: NCX block impact in the readjusted human ventricular cardiomyocyte model, with electroneutral PMCA; Figure S7: Comparison of readjusted human ventricular cardiomyocyte and hiPSC-CM models; Table S1: Patient characteristics; Table S2: Action potential parameters (mean ± SEM) before and after superfusion with SEA0400 (10 µM) in rat ventricle, human ventricle and EHT; Supplemental Experimental and Computational Procedures.

## Abbreviations

APs	action potentials
APA	action potential amplitude
APD	action potential duration
APD_90_	action potential duration at 90% repolarization
CaT	calcium transient
CM	cardiomyocyte
Cs	cell shortening
EHT	engineered heart tissue
hiPSC-CM	human-induced pluripotent stem cell-derived cardiomyocyte
I_Ca_	calcium current
I_K1_	inwardly rectifying potassium current density
I_Kr_	rapidly activating potassium current
KO	knock-out
LV	left ventricle
NCX	sodium calcium exchanger
PMCA	plasma membrane Ca^2+^-ATPase
TMCs	time-matched controls
