# Supplementary material for: Regulation of APD and Force by the Na+/Ca2+ Exchanger in Human-Induced Pluripotent Stem Cell-Derived Engineered Heart Tissue
Source: Cells. 2022 Aug 5;11(15):2424. doi: 10.3390/cells11152424 (PMC9368200; doi:10.3390/cells11152424)
Supplement: Supplementary file 1 [file cells-11-02424-s001.zip › cells-1763259-supplementary.pdf]

Figure S1: RNA expression of NCX

Mean values and SD for RNA expression of NCX isoforms 1-3 (NCX1, NCX2 and NCX3) in human left ventricle (human LV) and EHT.* indicates significance vs. human LV.

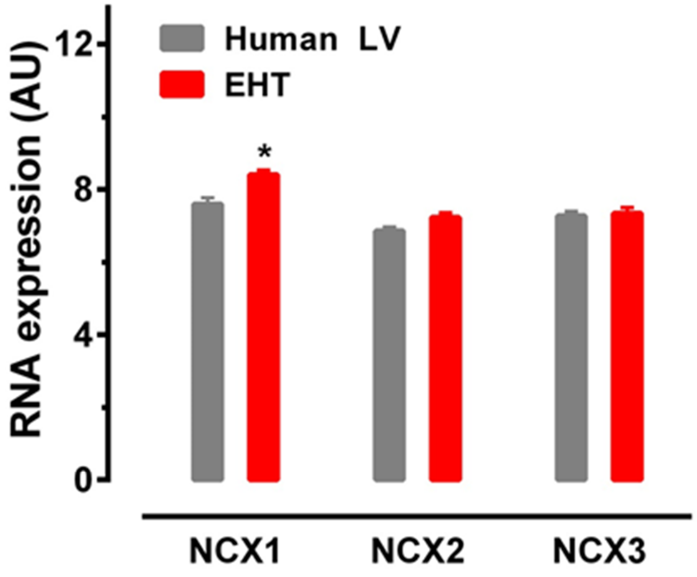

Figure S2: NCX currents in cardiomyocytes from human ventricle and EHT culture divided by different patients and different batches

(**A**) Mean values ± SEM or single current for NCX current density in CMs from human ventricle divided by different patients. (**B**) Mean values ± SEM for NCX current density in CMs from EHT divided by different batches.

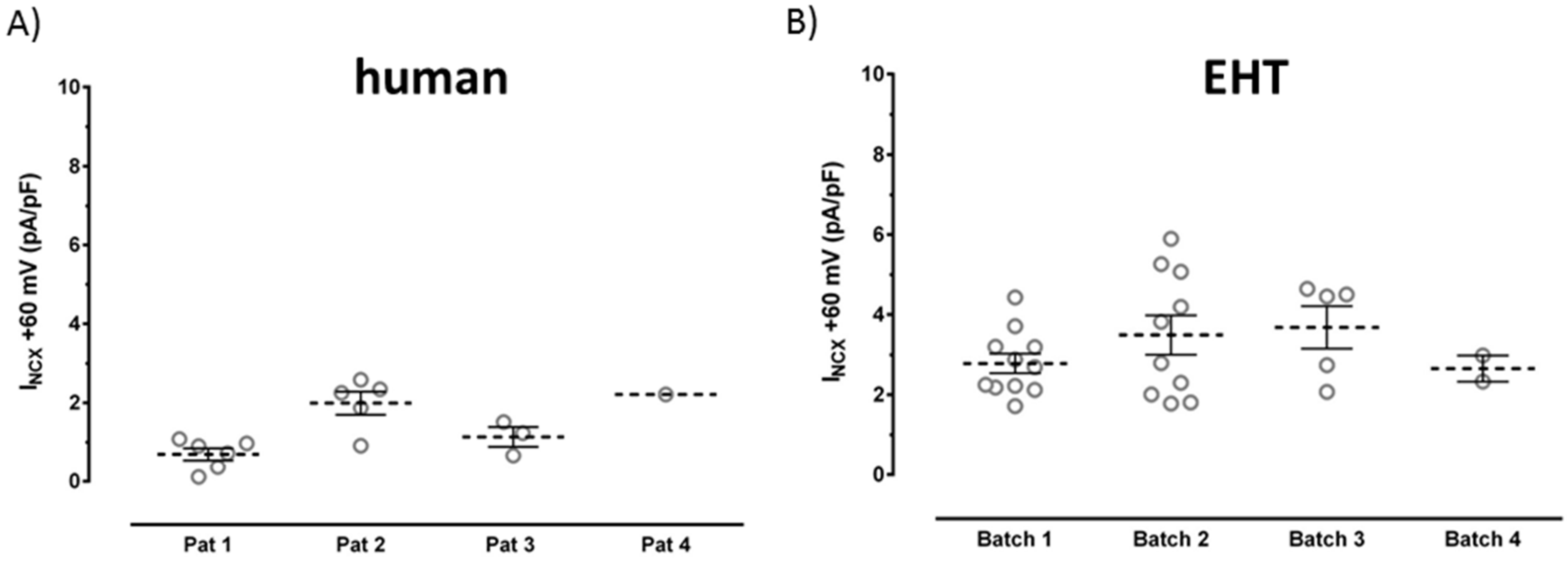
A)
human
I_{NCX} +60 mV (pA/pF)
10
8
6
4
2
0
Pat 1
Pat 2
Pat 3
Pat 4
B)
EHT
I_{NCX} +60 mV (pA/pF)
10
8
6
4
2
0
Batch 1
Batch 2
Batch 3
Batch 4

Figure S3: Effect of SEA0400 on calcium current (I_{Ca}) in EHT

Mean values ± SEM for time courses of calcium current (I_{Ca}) densities given as time-matched control (TMC) and exposed to 10 µM SEA0400 (SEA) at -120 s, 0 s (exposure of cells with SEA0400) and +120 s. n indicates number of measurements.

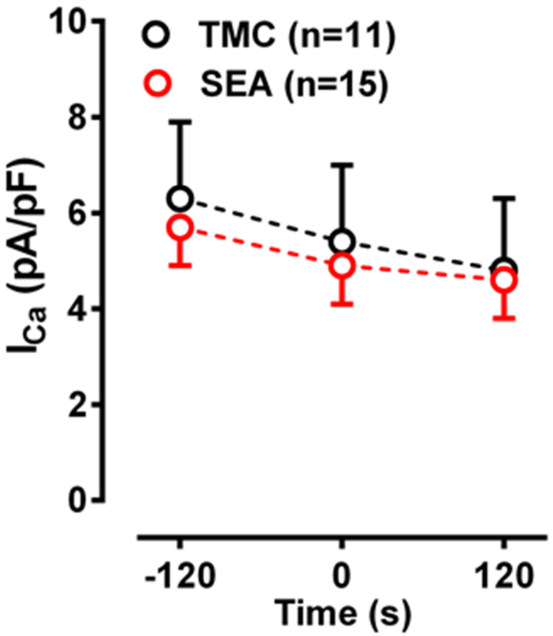

TMC (n=11)
SEA (n=15)
10
8
6
4
2
0
I_{Ca} (pA/pF)
-120
0
120
Time (s)

Figure S4: Comparison of NCX block impact in the original and readjusted rat ventricular cardiomyocyte models

The principal outputs: membrane voltage (**A**) and calcium transient (**B**) with different degrees of NCX block. (**C-D**) NCX current is slightly weaker and PMCA current slightly stronger in the readjusted model. (**E-F**) The original model develops spontaneous store overload-triggered action potential abnormalities at 50% NCX block, whereas the readjusted model copes with this degree of NCX block. However, the calcium transient is substantially larger also in the readjusted model (**B**, left panel, purple line). Fractions of Ca^{2+} removal SERCA = [88.2, 87.6]%, NCX = [10.1, 9.4]%, and PMCA = [1.7, 3.0]%, as well as PMCA-NCX ratio = [16.5, 32.5]% in the original and readjusted models, respectively.

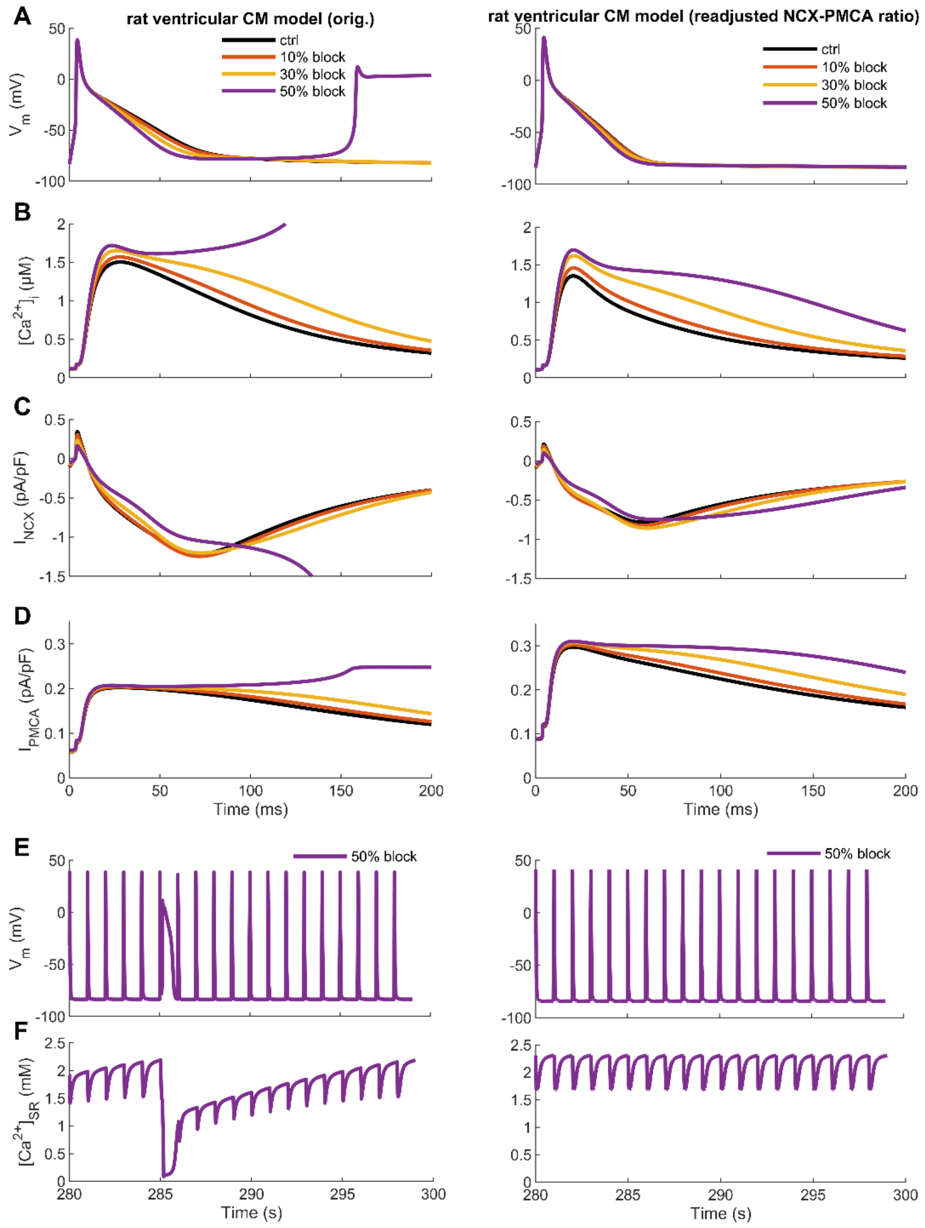

A
rat ventricular CM model (orig.)
rat ventricular CM model (readjusted NCX-PMCA ratio)
ctrl
10% block
30% block
50% block
V_m (mV)
B
[Ca2+]_i (μM)
C
I_NCX (pA/pF)
D
I_PMCA (pA/pF)
Time (ms)
E
50% block
V_m (mV)
F
[Ca2+]_SR (mM)
Time (s)

Figure S5: Comparison of NCX block impact in the original and readjusted human ventricular cardiomyocyte models

Membrane voltage (**A**), calcium transient (**B**), NCX current (**C**) and PMCA current (**D**) with different degrees of NCX block. (**B**) The original model predicts intracellular calcium overload (**B**, left panel, purple line; peaking at 6.7 µM), which causes action potential abnormalities (**A**, left panel, purple line). Whereas the readjusted model copes with this degree of NCX block. In the original model, the PMCA was in physiological terms non-existent (**D**, left panel, insert), almost three magnitudes smaller than in the readjusted model. Fractions of Ca^{2+} removal SERCA = [77.9, 82.5]%, NCX = [22.1, 11.9]%, and PMCA = [0.0071, 5.6]% in the original and readjusted models, respectively.

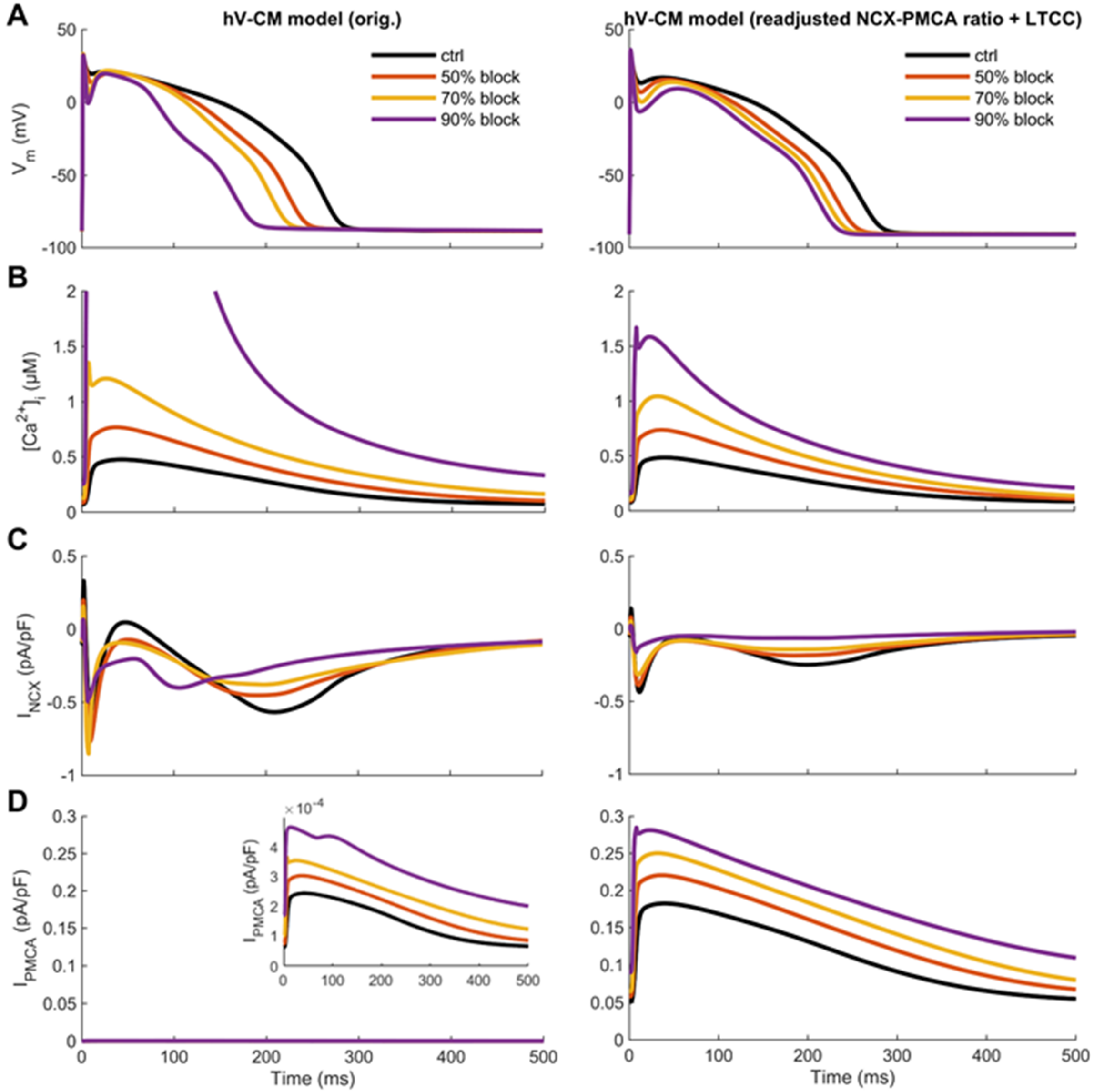

A
hV-CM model (orig.)
hV-CM model (readjusted NCX-PMCA ratio + LTCC)
ctrl
50% block
70% block
90% block
V_m (mV)
B
$[Ca^{2+}]_i$ (µM)
C
I_{NCX} (pA/pF)
D
I_{PMCA} (pA/pF)
$\times 10^{-4}$
Time (ms)

Figure S6: NCX block impact in the readjusted human ventricular cardiomyocyte model, with electroneutral PMCA

Membrane voltage (**A**), calcium transient (**B**), NCX current (**C**) and PMCA current (**D**) with different degrees of NCX block.

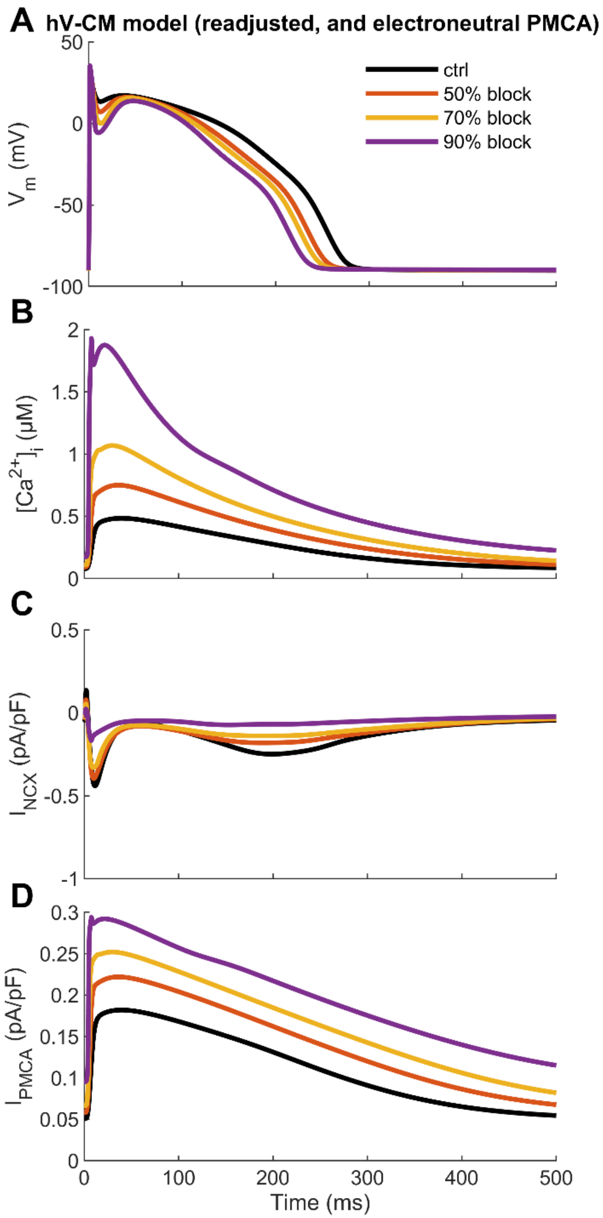

A
hV-CM model (readjusted, and electroneutral PMCA)
ctrl
50% block
70% block
90% block
V m (mV)
50
0
-50
-100
B
[Ca2+]i (µM)
2
1.5
1
0.5
0
C
INCX (pA/pF)
0.5
0
-0.5
-1
D
IPMCA (pA/pF)
0.3
0.25
0.2
0.15
0.1
0.05
0
0
100
200
300
400
500
Time (ms)

Figure S7: Comparison of readjusted human ventricular cardiomyocyte and hiPSC-CM models

The principal outputs: membrane voltage (**A**), calcium transient (**B**), and active tension (**C**). Differences in ion current, as result of 1.5-fold I_{Ca} density (**D**), 4-fold I_{Kr} density (**E**), and 0.5-fold I_{K1} density (**F**), as well as, a 2.5-fold stronger NCX (**G**).

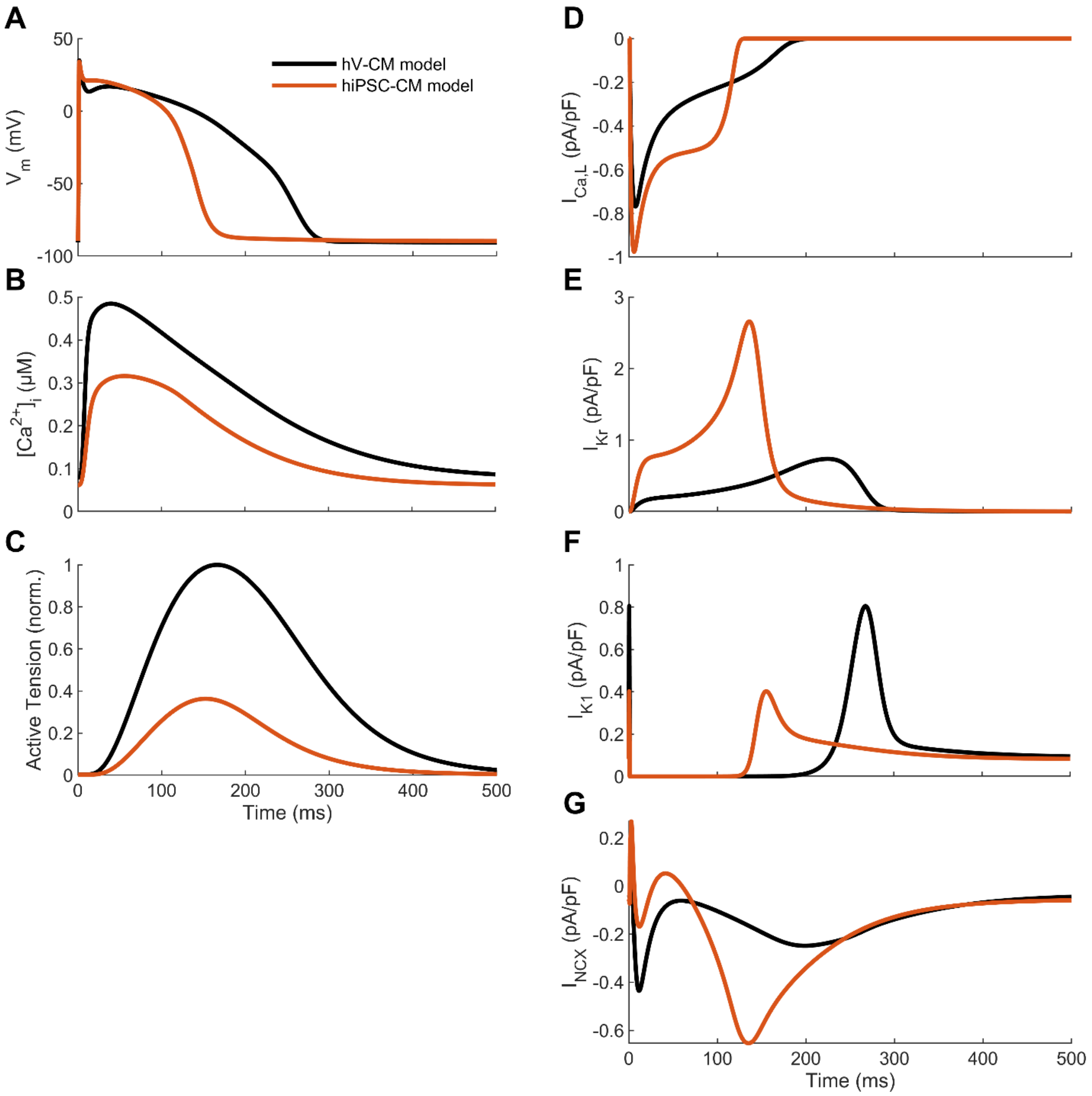

A
B
C
D
E
F
G
hV-CM model
hiPSC-CM model
V_m (mV)
[Ca2+]_i (μM)
Active Tension (norm.)
I_Ca,L (pA/pF)
I_Kr (pA/pF)
I_K1 (pA/pF)
I_NCX (pA/pF)
Time (ms)
Time (ms)

Table S1: Patient characteristics

	Overall
**Patients (n)**	13
Gender (m/f)	12/1
Age at surgery (mean ± SEM in years)	53.7±4.3
BMI (mean ± SEM in kg/m^2)	26.3±0.7
**Indication for surgery**	
Advanced heart failure by coronary artery disease (n)	6
Advanced heart failure by dilated cardiomyopathy (n)	4
Valve disease (n)	3
**Cardiovascular comorbidity**	
Arterial hypertension (n)	5
Diabetes mellitus (n)	3
Hyperlipoproteinemia (n)	6
**Echocardiography data**	
Left ventricular ejection fraction (mean ± SEM in %)	27.5±4.7
**Cardiovascular medication**	
β-blockers (n)	13
ACE-inhibitors/AT_1-blockers/ARNI (n)	11
Diuretics (n)	10
Ca^{2+}-channel-blockers (n)	2
Digitalis (n)	0
Nitrates (n)	0
Lipid-lowering drugs (n)	6

Table S2: Action potential parameters (mean±SEM) before and after superfusion with SEA0400 (10 µM) in rat ventricle, human ventricle and EHT

Parameter	Rat (n=7)		Human LV (n=6)		EHT (n=7)	
	Basal	SEA0400 (10 µM)	Basal	SEA0400 (10 µM)	Basal	SEA0400 (10 µM)
RMP (mV)	-77.8±1.1	-76.6±1.6	-73.8±2.8	-71.8±3.9	-79.2±2.6	-78.4±2.2
APA (mV)	97.8±1.9	94.9±2,7	102.9±4.9	99.4±6.9	112.3±3.8	110.9±3.2
V_{max} (mV/s)	153.6±26	193.9±52.9	139.7±44.7	133.2±39.7	304.6±96.3	303.9±88.2
APD_{50} (ms)	16.6±1.1	16.3±1.5	235.3±18.6	224.7±16.9	191.1±13.6	128.6±15.4
APD_{90} (ms)	54.4±3.9	48.9±4.1	319.9±22	305.4±20.3	257.7±16.3	197.3±18.8

Supplemental Experimental and Computational Procedures

Microarray analysis

Microarray analyses were performed following previously described protocols [1,2]. Briefly, after culturing period EHTs were digested with Proteinase K (Qiagen 19133) at 56 °C for 10 minutes. Isolation of RNA was performed with the RNeasy® mini kit (Qiagen 74104). The Illumina GenomeStudio V2011.1 Platform (Gene Expression Module 1.9.0) was used to process array data. Statistical analyses were performed with the software Partek Genomic Suite 6.7.

Readjusting the SERCA-NCX-PMCA calcium removal fractions in the rat ventricular cardiomyocyte model

The readjusted parameters and their modified values are given in the Methods section. Here, we describe in more detail, what literature was used as a basis for those modifications, and how.

Contrary to the common, perhaps the most common, paradigm Ca^{2+} recycling contributions in murine CMs (SERCA ~90%, NCX extrudes ~10%, PMCA ~1%), there is a substantial body of experimental data suggesting that PMCA can remove Ca^{2+} at a rate of 30% of that of NCX [3–5]. In the original implementation of the rat ventricular CM model [6], the fractions were actually pretty close to that range SERCA 88.2%, NCX 10.1%, and PMCA 1.7%. Thus, no dramatic modifications were needed. We decreased the maximum exchange rate of NCX by 20% and increased the maximum pump rate of PMCA by 50%. In addition, the conductance of background Ca^{2+} current was decreased by 20%, to restore the sarcoplasmic reticulum [Ca^{2+}] to its original range. With these modifications, the Ca^{2+} recycling contributions SERCA, NCX, and PMCA were [87.6, 9.4, 3.0] %, respectively. Importantly, the readjusted rat ventricular CM model appeared a bit more robust than the original one, capable of

handling a 50% NCX block (Figure S4). However, at higher NCX block ratios, even this readjusted model had intracellular Ca^{2+} overload and consequent AP abnormalities (data not shown), similar to the original model at 50% NCX block (Figure S4, left column, panels A and F).

Readjusting the SERCA-NCX-PMCA calcium removal fractions in the human ventricular cardiomyocyte model

To our knowledge, a thorough analysis of SERCA-NCX-PMCA Ca^{2+} removal fractions for human ventricular CMs has not been published. Such work has, however, been carried out with human atrial CMs, reporting SERCA-NCX-PMCA Ca^{2+} removal fractions of 66%, 25%, and 9% respectively by Voigt et al. [7] (Online-Figure IIID in the original publication). Furthermore, there is one partial data set by Maier et al. [8] (Fig. 3 in the original publication) suggesting that while the frequency dependence of SERCA contribution to Ca^{2+} removal fractions in human ventricular and atrial CMs are quite different, at the pacing frequency of 0.75 Hz they are equal. The original Maier et al. data is shown in Figure S8A.

Based on these two data sets, we estimated the SERCA-NCX-PMCA Ca^{2+} removal fractions in human ventricular CMs at the other pacing frequencies, such as 1 Hz. First, we used the Voigt et al. that showed SERCA contribution to be 66% at 0.5 Hz pacing. Figure S8B shows the Maier et al. atrial data (red dashed line), when it has been shifted to match the Voigt et al. data at 0.5 Hz pacing, and the ventricular data (blue dashed line), when it has been shifted alike to preserve interject at 0.75 Hz. Furthermore, we needed to acknowledge that Voigt et al. did not use a standard current pulse stimulus in their experiments. Instead, they used, according to their own in-house protocol, a voltage clamp as stimulus. We hypothesized that their protocol might overestimate NCX contribution to Ca^{2+} removal. To test our hypothesis, we replicated the experiments in simulations, using our human atrial CM model [9]. Indeed, our simulation results suggested that during 0.75 Hz pacing, using a normal current stimulus the SERCA contribution to Ca^{2+} removal would be as high as 82% (Figure S8C, red dashed line), while NCX and PMCA fractions would be 12% and 6%, respectively. Accordingly, the SERCA contribution in human ventricular CMs (Figure S8C, blue dashed line) was obtained by

shifting the data alike, to preserve the interject at 0.75 Hz. Figure S8D summarizes the data: Voigt et al. data as the lower limit and our estimation as the upper limit.

Next, we readjusted the Ca^{2+} removal fractions in the recently published human ventricular CM model [10]. In the original model version, the SERCA-NCX-PMCA fractions were [78, 22, 0.0071] %. That is, the PMCA fraction was negligible, with almost no impact on Ca^{2+} extrusion. To reduce NCX and dramatically increase PMCA fraction, we decreased the maximum exchange rate of NCX by 60% and the PMCA pump rate was increased by 740-fold (from 5e-04 to 0.37). This was quite a substantial modification to the model dynamics, therefore we also decreased the maximum permeability of L-type Ca^{2+} channel by -33%, to reset the sarcoplasmic reticulum [Ca^{2+}] to the original range. Furthermore, additional tuning of K^{+} current conductances was needed to reset the APD to the original range. We accomplished this by using global multiplier with a value of 0.73, to reduce the maximum conductance of all K+ currents by 27%. After these readjustments, the SERCA-NCX-PMCA fractions were [80, 13, 6.6] % at 0.75 Hz pacing, falling nicely within the range that we estimated based on the Maier et al. and Voigt et al. data (Figure S8D).

Supplemental References

[1] I. Mannhardt, K. Breckwoldt, D. Letuffe-Brenière, S. Schaaf, H. Schulz, C. Neuber, A. Benzin, T. Werner, A. Eder, T. Schulze, B. Klampe, T. Christ, M.N. Hirt, N. Huebner, A. Moretti, T. Eschenhagen, A. Hansen, Human Engineered Heart Tissue: Analysis of Contractile Force, Stem Cell Reports. 7 (2016) 29–42. https://doi.org/10.1016/j.stemcr.2016.04.011.

[2] Z. Iqbal, D. Ismaili, B. Dolce, J. Petersen, H. Reichenspurner, A. Hansen, P. Kirchhof, T. Eschenhagen, V.O. Nikolaev, C.E. Molina, T. Christ, Regulation of basal and norepinephrine-induced cAMP and ICa in hiPSC-cardiomyocytes: Effects of culture conditions and comparison to adult human atrial cardiomyocytes, Cell. Signal. 82 (2021) 109970. https://doi.org/10.1016/J.CELLSIG.2021.109970.

[3] N. Negretti, S.C. O'Neill, D.A. Eisner, The relative contributions of different intracellular and sarcolemmal systems to relaxation in rat ventricular myocytes, Cardiovasc. Res. 27 (1993) 1826–1830. https://doi.org/10.1093/CVR/27.10.1826.

[4] H.S. Choi, D.A. Eisner, The role of sarcolemmal Ca2+-ATPase in the regulation of resting calcium concentration in rat ventricular myocytes, J. Physiol. 515 (1999) 109–118. https://doi.org/10.1111/J.1469-7793.1999.109AD.X.

[5] H.S. Choi, D.A. Eisner, The effects of inhibition of the sarcolemmal Ca-ATPase on systolic calcium fluxes and intracellular calcium concentration in rat ventricular myocytes, Pflügers Arch. 1999 4376. 437 (1999) 966–971. https://doi.org/10.1007/S004240050868.

[6] S. Gattoni, Å.T. Røe, M. Frisk, W.E. Louch, S.A. Niederer, N.P. Smith, The calcium–frequency response in the rat ventricular myocyte: an experimental and modelling study, J. Physiol. 594 (2016) 4193–4224. https://doi.org/10.1113/JP272011.

[7] N. Voigt, N. Li, Q. Wang, W. Wang, A.W. Trafford, I. Abu-Taha, Q. Sun, T. Wieland, U. Ravens, S. Nattel, X.H.T. Wehrens, D. Dobrev, Enhanced sarcoplasmic reticulum Ca 2+ Leak and increased Na +-Ca 2+ exchanger function underlie delayed afterdepolarizations in

patients with chronic atrial fibrillation, Circulation. 125 (2012) 2059–2070. https://doi.org/10.1161/CIRCULATIONAHA.111.067306.

[8] L.S. Maier, P. Barckhausen, J. Weisser, I. Aleksic, M. Baryalei, B. Pieske, Ca2+ handling in isolated human atrial myocardium, Am. J. Physiol. - Hear. Circ. Physiol. 279 (2000) 952–958. https://doi.org/10.1152/AJPHEART.2000.279.3.H952/ASSET/IMAGES/LARGE/H40900143005.JPEG.

[9] L. Skibsbye, T. Jespersen, T. Christ, M.M. Maleckar, J. van den Brink, P. Tavi, J.T. Koivumäki, Refractoriness in human atria: Time and voltage dependence of sodium channel availability, J. Mol. Cell. Cardiol. 101 (2016). https://doi.org/10.1016/j.yjmcc.2016.10.009.

[10] F. Margara, Z.J. Wang, F. Levrero-Florencio, A. Santiago, M. Vázquez, A. Bueno-Orovio, B. Rodriguez, In-silico human electro-mechanical ventricular modelling and simulation for drug-induced pro-arrhythmia and inotropic risk assessment, Prog. Biophys. Mol. Biol. 159 (2021) 58–74. https://doi.org/10.1016/J.PBIOMOLBIO.2020.06.007.
